# Supplementary material for: iRGD synergizes with PD-1 knockout immunotherapy by enhancing lymphocyte infiltration in gastric cancer
Source: Nat Commun. 2019 Mar 22;10:1336. doi: 10.1038/s41467-019-09296-6 (PMC6430780; doi:10.1038/s41467-019-09296-6)
Supplement: Supplementary file 1 — Supplementary Information [file 41467_2019_9296_MOESM1_ESM.pdf]

iRGD synergizes with PD-1 knockout immunotherapy by enhancing lymphocyte infiltration in gastric cancer

Ding et al.

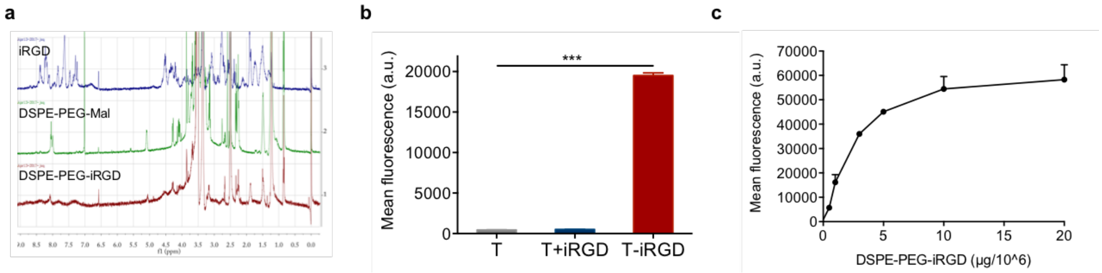

Supplementary Figure 1 Cell surface modification with DSPE-PEG-iRGD. a. ^{1}H NMR spectrum of iRGD, DSPE-PEG-Mal and DSPE-PEG-iRGD. **b.** Mean fluorescence intensity of T cells alone (grey) and the cells incubated with iRGD-FAM (blue) and DSPE-PEG-iRGD-FAM (red). Student's *t* test. ***p < 0.001. **c.** Analysis of mean fluorescence intensity of cells treated with different ratios of DSPE-PEG-iRGD. Data represent mean ± s.e.m.; n = 3.

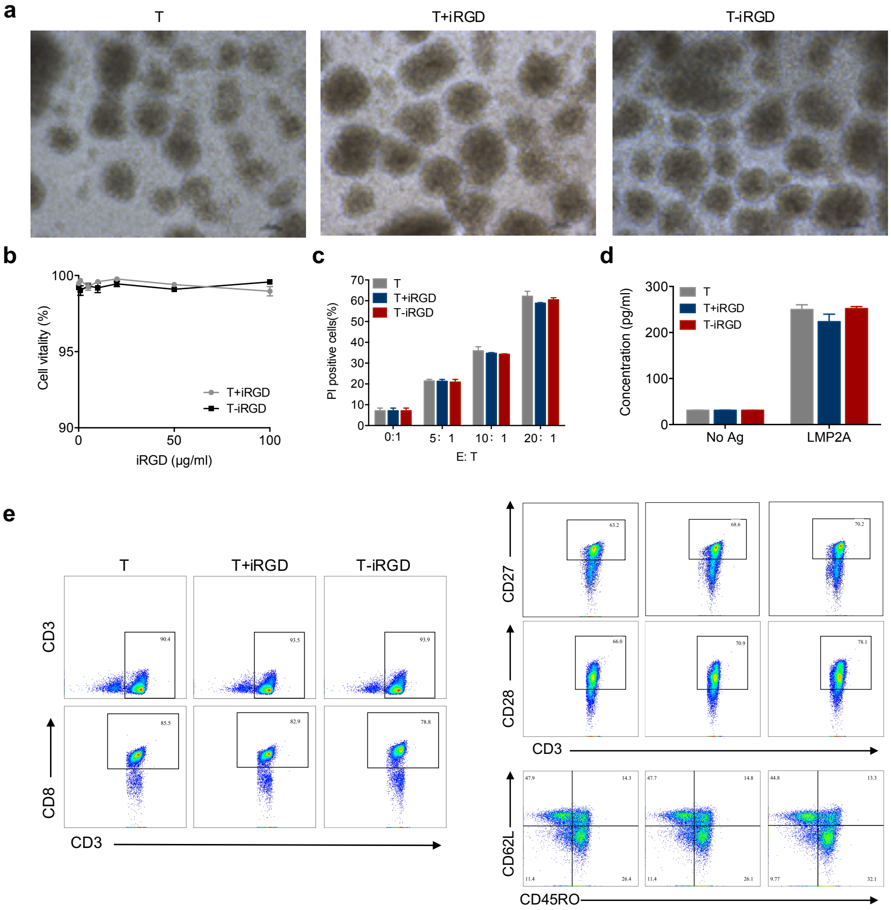

Supplemetary Figure 2 Influence of DSPE-PEG-based modification on T cells *in vitro*. a. No morphological change was seen in T, T+iRGD and T-iRGD group. *In vitro* cultured activated lymphocytes were exposed to iRGD or DSPE-PEG-iRGD solutions at the concentration of 5 μg ml^{-1} for 24 hours, the morphological change was analyzed by optical microscopy. Scale bar, 100 μm**. b.** Vitality of cells after exposure to iRGD or DSPE-PEG-iRGD solutions with different concentrations. **c-e.** Cytotoxicity, cellular response and phenotype of EBV-LMP2A-specific CTLs of different format. LMP2A-specific CTLs were induced by co-culturing of HLA-A24 positive human PBMC with HLA-A24-restricted LMP2A peptide (TYGPVFMCL) loaded DCs in the presence of IL-2, IL-7 and IL-15 for 14 days. And then, EBV-LMP2A-CTLs were incubated with iRGD or DSPE-PEG-iRGD solutions at the concentration of 5 μg ml^{-1} for 30 minutes and analyzed. LMP2A-specific CTLs of different format were incubated with CFSE labeled HLA-A24 positive SNU719 cells at effector-to-target ratio (E:T) of 0:1, 5:1, 10:1, 20:1 and 40:1 respectively, PI was added 6 hours after incubation and the percentage of dead cells was analyzed by flow cytometry (**c**). CTLs were stimulated with LAM2A loaded autologous DCs for 20 hours, culture supernatant was collected and the cytokine level was analyzed by CBA Human IFN-γ kit (**d**). The phenotype of the in vitro cultured T cells of different format were analyzed by flow cytometry (**e**). Data represent mean ± s.e.m.; n = 3. Student's *t* test.

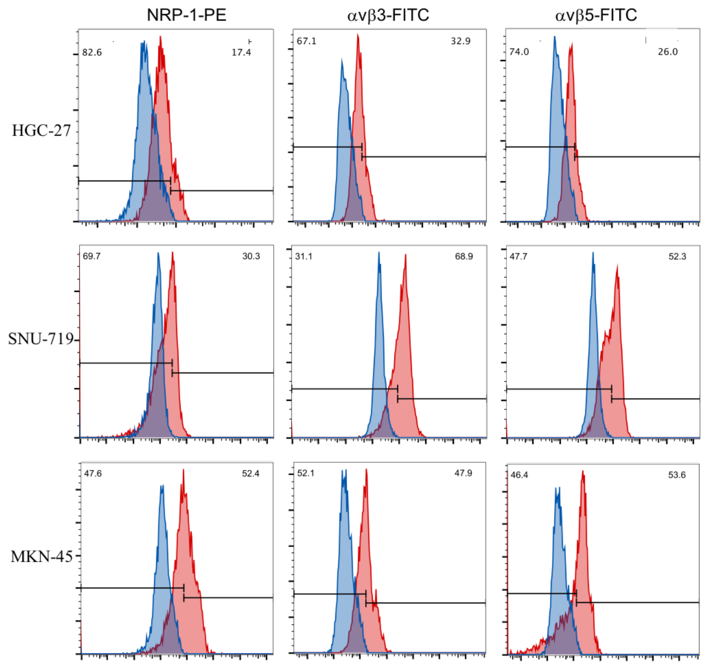

Supplementary Figure 3 Cell expression of αvβ3, αvβ5 and NRP-1. αvβ3, anti-αvβ5 and NRP-1 expression in tumor cells was determined by flow cytometry. Integrin αvβ3 was detected using FITC-conjugated mouse anti-human αvβ3 monoclonal antibody, and integrin αvβ5 was detected using FITC-conjugated mouse anti-human αvβ5 monoclonal antibody. The matched isotype control was FITC-conjugated mouse IgG1κ. NRP-1 was detected using PE-conjugated mouse anti-human NRP-1 monoclonal antibody and an isotype control.

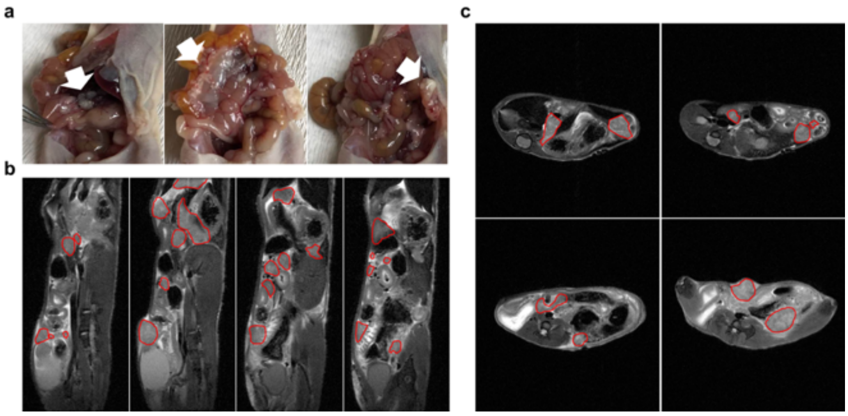

Supplementary Figure 4 The establishment of peritoneal metastasis tumor model. 10^6 MKN45 cells were injected intraperitoneally and 20 days later, the formation of tumor nodules were evaluated by both dissection (**a**) and T2-weighted MR imaging (**b, c**). The white arrow and the red circle indicate the tumor nodules.

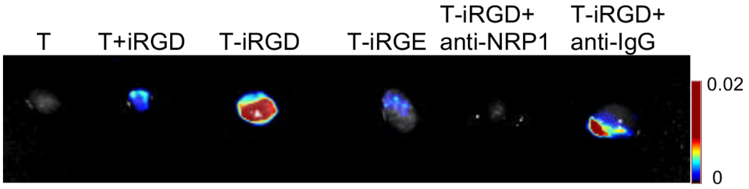

Supplementary Figure 5 iRGD-mediated *in vivo* tumor penetration of T cells in intraperitoneal tumor model. Mice bearing intraperitoneal tumor were treated with 50ug anti-NRP-1 antibody or control sheep anti-IgG 15 min prior to T cells injection, tumor nodules were analyzed 3 h post T cells injection.

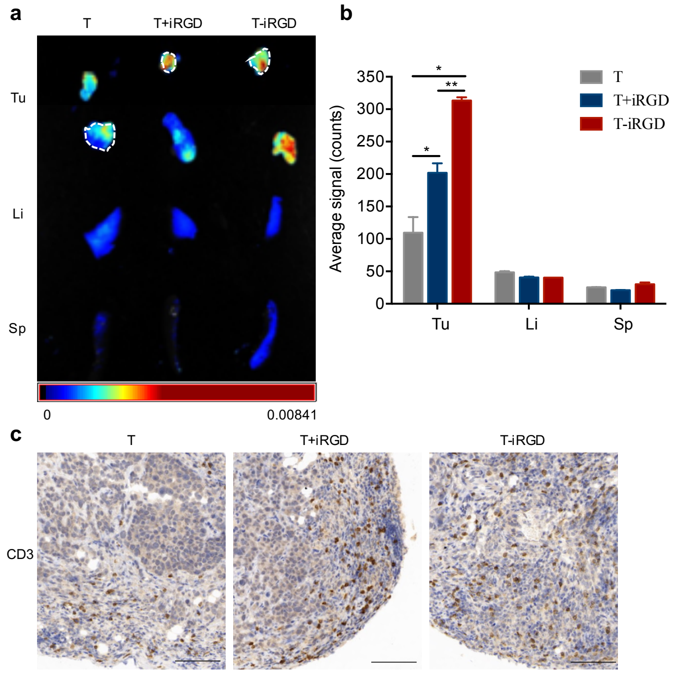

Supplementary Figure 6 Enhanced tumor specific delivery of lymphocytes 24 h post intraperitoneal injection. a. *Ex vivo* images of tumor, liver and spleen at 24 h post intraperitoneal T cells injection. top layer: small tumor nodules (diameter < 3mm); second layer: big tumor nodules (diameter > 3mm). White dashed lines, peritoneal tumors. **b.** Quantification of the fluorescent signal in tumors and control organs at 24 h post lymphocytes injection. Data are represented as mean ± s.e.m.; n = 3. Student's *t* test. $^{**}p < 0.05$, $^{*}p < 0.01$. **c.** Immunohistochemistry analysis of resected tumor nodules at 24 h post intraperitoneal injection. T cells were stained with anti-CD3. Scale bar, 100 μm.

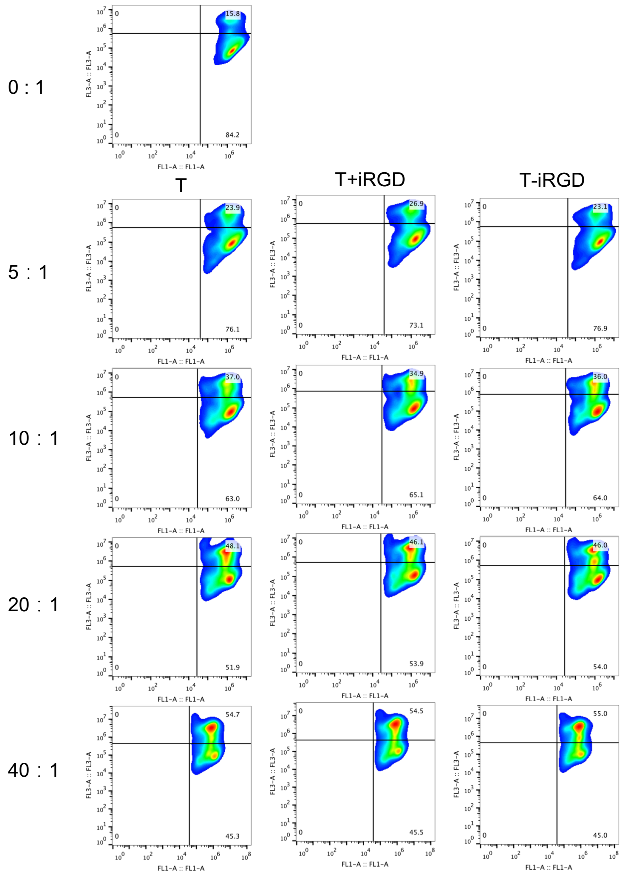

Supplementary Figure 7 Cytotoxicity of iRGD modified T cell on MKN45 in vitro. Activated T cells of different format were incubated with CFSE labeled MKN45 cells at effector-to-target ratio (E:T) of 0:1, 5:1,10:1,20:1 and 40:1 respectively, PI was added 6 hours after incubation and the percentage of dead cells was analyzed by flow cytometry.

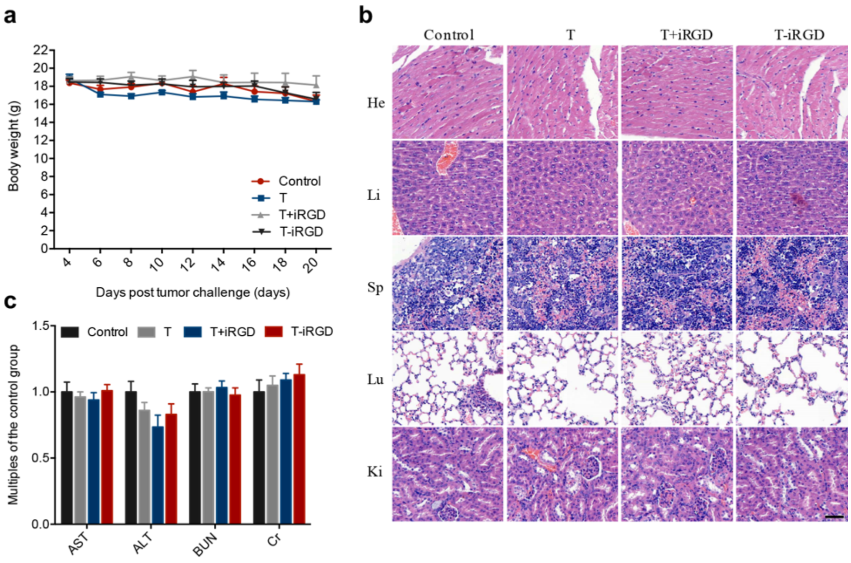

Supplementary Figure 8 Safety profile evaluation of intraperitoneal T cells injection. a. Body weight change of mice treated by intraperitoneal injections of PBS, T, T+iRGD or T-iRGD. **b.** Main organs were dissected for H&E staining. Scale bar, 100 μm. **c.** Assessment of liver and kidney function at the end of treatment. Data are represented as mean ± s.e.m.; n = 8. Student's *t* test. He, Heart; Li, Liver; Sp, Spleen; Lu, Lung; Ki, Kidney; AST, Aspartate aminotransferase; ALT, Alanine aminotransferase; BUN, Blood urea nitrogen; Cr, Creatinine.

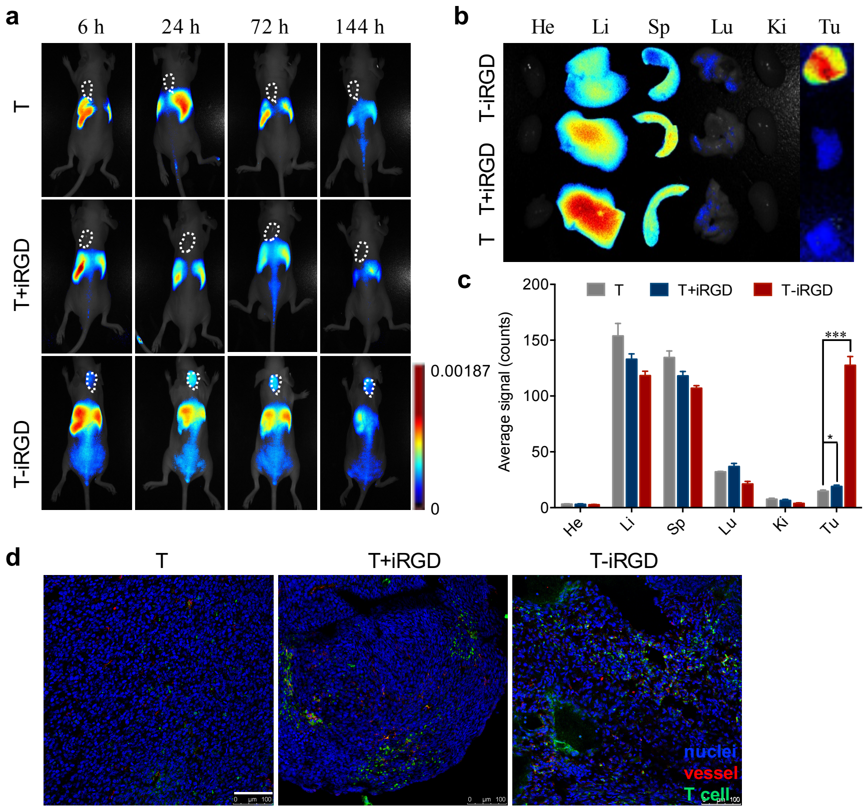

Supplementary Figure 9 Tumor target infiltration of iRGD modified lymphocytes in a SNU719 subcutaneous tumor model. a. *In vivo* imaging of SNU719 tumor-bearing mice at 6, 24, 72 and 144 h after intravenous injection of DiR labeled T cells. White dashed lines, subcutaneous tumors. **b.** *Ex vivo* imaging of tumors and other organs at 144 h after T cells injection. **c.** Semiquantification of T cells biodistribution in mice organs collected at 144 h post T cells injection. Results are expressed as mean fluorescence intensity ± s.e.m.; n = 5. Student's *t* test. *p < 0.05, ***p < 0.001. **d.** Confocal images of frozen tumor sections at 24 h post T cells transfusion. T cells were labeled with CFSE before injection. Tumor blood vessels were labeled with CD31.T cells, green; vessel, red; nucleus, blue. Scale bar, 100 μm.

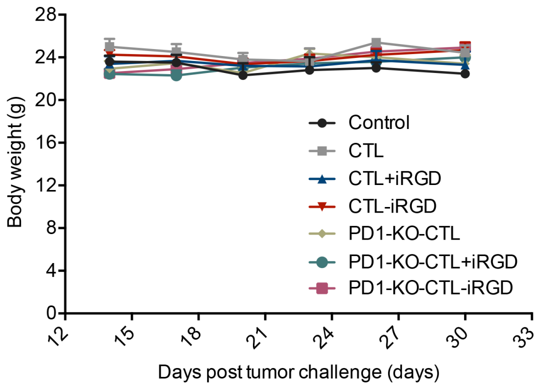

Supplementary Figure 10 Body weight change of SNU719 tumor-bearing mice that received the indicated treatments. Data are represented as mean ± s.e.m.; n = 7.

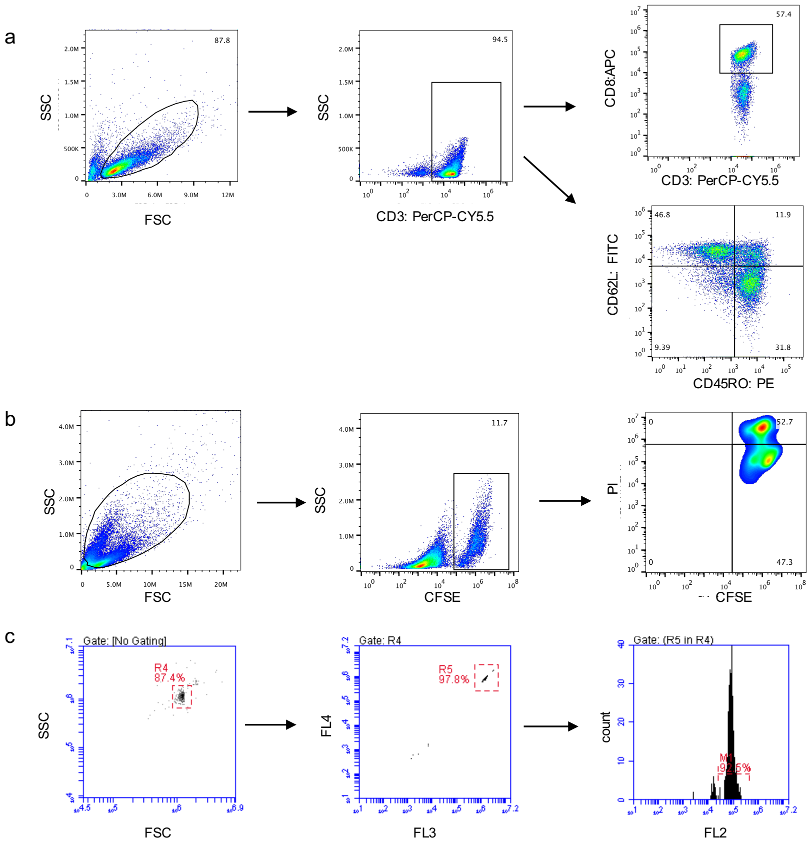

Supplementary Figure 11 Representative Sequential Gating Strategies for Flow Cytometry Analysis. a. Gating strategy to identify the phenotype of the in vitro cultured T cells presented in Supplementary Figure 2e. The gating strategy depicted here is also applied to experiments from which data is shown in Figure 1c-e, Supplementary Figure 2b and Supplementary Figure 3. **b.** Gating strategy to determine the percentage of dead cell presented in Supplementary 2c and Supplementary 7. **c.** Gating strategy to determin the IFN-γ level presented in Supplementary 2d.
